# Single coronary ostium in a patient with quadricuspid aortic valve combined with aneurysmal ascending aortic dilatation

**DOI:** 10.1186/s13019-017-0622-4

**Published:** 2017-07-24

**Authors:** Do Yeon Kim, Hwan Wook Kim

**Affiliations:** Department of Thoracic and Cardiovascular Surgery, Seoul St. Mary’s Hospital, College of Medicine, The Catholic University of Korea, 222 Banpo-daero, Seocho-gu, Seoul, 137-701 Republic of Korea

**Keywords:** Quadricuspid aortic valve, Aneurysmal ascending aortic dilatation, Single coronary ostium

## Abstract

**Background:**

The presence of a fourth aortic valve cusp (quadricupsid aortic valve) is a rare congenital malformation and is often accompanied by other anomalies of the adjacent cardiovascular structures. Among these concomitant anomalies, simultaneous association of both a single coronary ostium *and* aneurysmal ascending aortic dilation in combination with the quadricupsid aortic valve has not been reported yet.

**Case presentation:**

We experienced the case of a 56-year-old female patient presenting as aortic regurgitation resulted from malcoaptation of quadricupsid aortic valve. The patient had also accompanying aneurysmal ascending aortic dilatation and coronary ostial anomaly. Surgical correction (aortic valve replacement with mechanical devices and supracoronary aortic replacement with prosthetic graft) was performed without any complications.

**Conclusions:**

The technological development of preoperative imaging studies enable the physician to encounter the quadricuspid aortic valve with other associated malformations more often unlike previous reports. With review on the quadricuspid aortic valve, we discussed a surgical considerations for the treatment of this anomaly.

**Electronic supplementary material:**

The online version of this article (doi:10.1186/s13019-017-0622-4) contains supplementary material, which is available to authorized users.

## Background

Quadricuspid aortic valve (QAV) is a rare congenital valvular abnormality, reported in only 2 cases of 6000 autopsies study with an incidence of 0.033% [1], but the incidence is probably underestimated. Moreover, it is even rarer for QAV to be combined with either single coronary ostium or aneurysmal ascending aortic dilatation [2]. Advances in noninvasive diagnostic techniques enable us to reveal precise anatomical configurations of this morphology along with other accompanying anomalies preoperatively.

We report the case of an adult female patient treated by surgery (aortic valve replacement with supracoronary ascending aortic replacement) for the regurgitant QAV with both concomitant single coronary ostium *and* aneurysmal ascending aortic dilatation. We include a literature review on QAV. To the best of our knowledge, the combination of these two accompanying anomalies (single coronary ostium *and* aneurysmal ascending aortic dilatation) in conjunction with QAV has not been reported previously.

## Case presentation

**Fig. 1:**
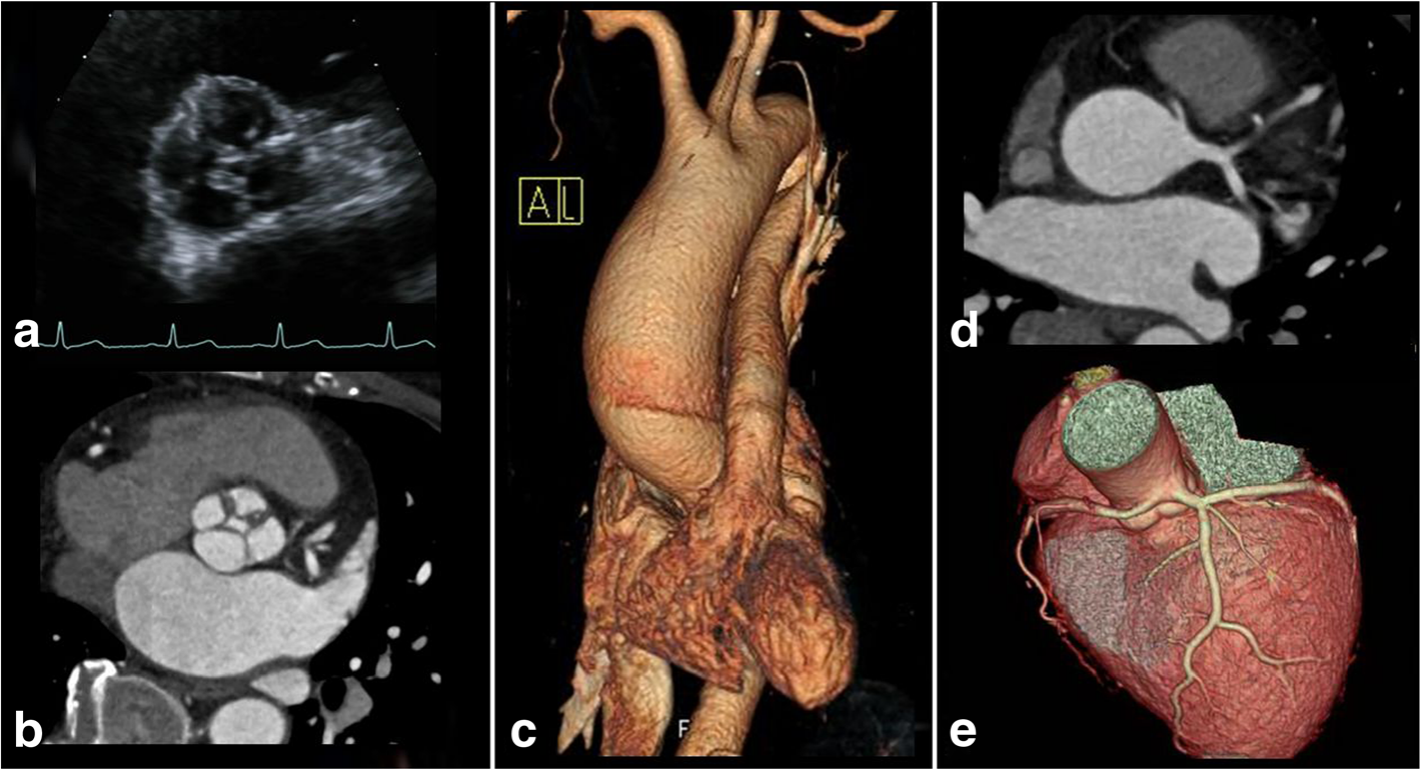
**a.** Transthoracic echocardiography showing a quadricuspid aortic valve with an ‘X-shaped’ commissural contour in the diastole. **b.** Computed tomographic angiography showing an aortic valve consisting of four symmetrically-sized cusps and a central coaptation defect resulting in central aortic regurgitation. **c.** Three-dimensional reconstruction imaging of computed tomography showing aneurysmal ascending aortic dilatation (maximum diameter = 45 mm) with preservation of the normal dimensions of the aortic root. **d.** and **e.** Volume-rendered multidetector computed tomography showing the spatial and anatomical relationships of the aortic cusps and the oval-shaped, single united coronary ostium

**Fig. 2:**
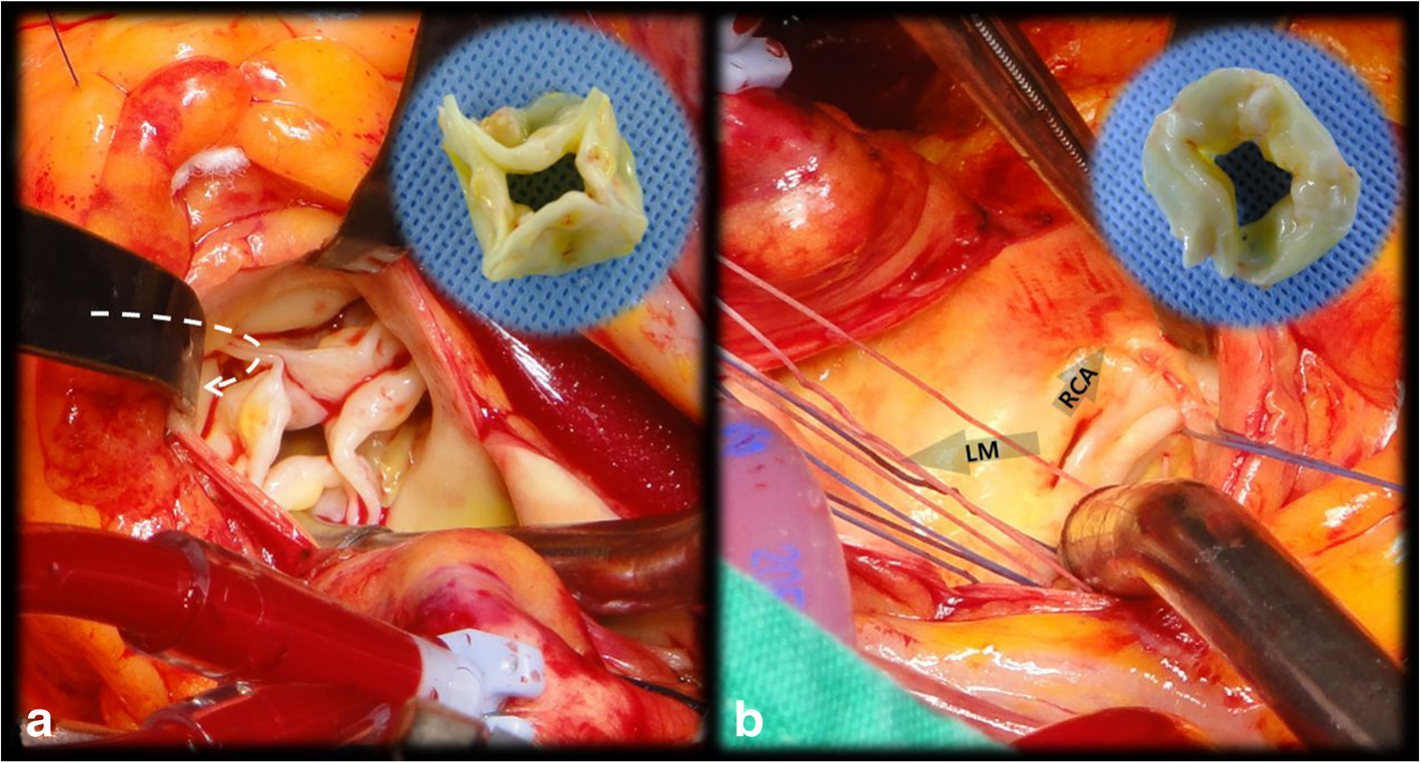
**a.** Intraoperative exploration showing a quadricuspid aortic valve with wrinkling fibrotic thickening of the free margin and commissural fusion. Both coronary arteries originated from only one cusp (see *arrow*). **b.** Intraoperative view of the slit-shaped, ovoid single united coronary ostium and imaginary directions of the left main coronary artery (LM) and the right coronary artery (RCA). Two inner circular figures showing each side of the resected quadricuspid aortic valve

A 56-year-old Korean female was referred for progressive symptoms characterized by dyspnea on exertion. A 4/6 diastolic murmur along the left sternal border following systolic murmur was heard upon cardiac auscultation; and mild cardiomegaly was detected on a chest roentgenogram. Upon admission of the patient, transthoracic echocardiography revealed a grade III aortic regurgitation, which was caused by an anomaly of the aortic valve characterized by an ‘X’-shaped commissural morphology during diastole (Fig. 1a, Additional file 1). A left ventricular hypertrophy with a dilated left ventricular cavity (LV mass index: 120 g/m2, LV end-diastolic dimension: 73 mm) was also detected. Electrocardiogram-gated computed tomographic aortography revealed a QAV with four symmetrically-sized cusps and a central coaptation failure with a regurgitation orifice (Fig. 1b). Furthermore, aneurysmal ascending aortic dilatation (maximum diameter = 45 mm), with preservation of the normal dimensions of the aortic root, is demonstrated (Fig. 1c). In addition, the ostium of the left coronary artery is juxtaposed with that of the right coronary artery, resulting in a single united ostial formation in the left coronary sinus (despite the normal course of coronary arteries presenting without stenosis) (Fig. 1d). Volume rendering of 3-dimensional reconstruction imaging showed the spatial and anatomical relationships of the ostia of two coronary arteries, originating from the oval-shaped, single united ostium, with adjacent structures (Fig. 1e). Under the diagnosis of dysfunctional QAV with concomitant single coronary ostium and separate aneurysmal ascending aortic dilatation, surgical correction (aortic valve replacement with mechanical devices and supracoronary aorta replacement with prosthetic graft) was performed. At surgery, the aortic valve was confirmed to be quadricuspid (Type A in anatomical classification by Hurwitz, et al.), with wrinkling fibrotic thickening of the free margin and commissural fusion (Fig. 2a). Resection of QAV disclosed the oval-shaped, single ostium to both coronary arteries, as seen on the preoperative multidetector computed tomography (Fig. 2b). Hereby, the location of the accessory cusp was not specified; and another classification suggested by Nakamura, et al. was not applicable to this case. The patient was discharged without any complications 8 days postoperatively.

## Discussion

The QAV is believed to be a very rare aortic valve abnormality with an incidence of up to 0.043% in an echo studies [3]. Usually, QAV disease is accidentally found upon autopsy or is unexpectedly found upon surgery for aortic valve disease. However, in a review of patients undergoing aortic valve replacement for aortic regurgitation, an incidence of approximately 1% was reported [4]. Therefore, the incidence is probably underestimated.

For surgical correction of cardiovascular disease, the physician must be aware of the potential anatomical variations in structure. Two anatomical classifications of QAV have been addressed. According to the potential discrepancies in size between the 4 cusps, Hurwitz et al. have classified QAV into seven subtypes (A to G) which do not identify the positional information of the accessory cusp [1]. The QAV with 3 equal-sized cusps and 1 smaller cusp (Type B) was presented in Hurwitz’s study as being the most common type. Comparatively, using the location of the supernumerary cusp to reclassify the QAV into four subtypes (I to IV) have been suggested by Nakamura et al. [5]. The most frequent variation in Nakamura’s study was the QAV with the supernumerary cusp being located between the right coronary cusp and the noncoronary cusp (Type II). Though Nakamura’s classification could theoretically provide more straightforward surgical information than would Hurwitz’s one, the Nakamura’s classification has not been applied to some variants with single coronary ostium—as in our case.

The QAV usually appears as an isolated asymptomatic malformation without evidence of hemodynamic alterations or other associated heart defect, but has also been reported to present with other coexistent cardiac malformations—such as anomaly of the coronary artery system, pulmonary valve stenosis, ventricular septal defect, non-obstructive cardiomyopathy, and aneurysmal ascending aortic dilatation. Among these various cardiac anomalies, ‘anatomic variations’ in coronary arterial system is known as being the most common such anomaly (and one being of particular importance from a surgical point of view) [4]. Malposition of the coronary ostium has been reported in about 10% of QAV setting [6]. Therefore, it is crucial to be aware of the anatomic variations of coronary arterial system during aortic annular fixation of the valvular prosthesis. In addition, as aortic regurgitation resulting from coaptation failure is mainly clinical presentation in patients with QAV, retrograde cardioplegia needs to be weighed carefully in the regurgitant QAV combined single coronary ostium, especially for optimal myocardial protection.

Furthermore, the downward displacement of the aortic annulus is worthy of notice at the time of aortic valve replacement, especially within a QAV in which the supranumerary cusp is located between the right coronary and noncoronary cusps (Type II in Nakamura’s classification). In this type, sutures located around annulus between the right coronary and noncoronary cusps, could be a potential risk factor for injury to the conduction bundle where it passes the membranous septum between these two cusps, thereby resulting in complete conduction block [7]. In reality, this variation (Type II) was the most frequent type in Nakamura’s classification. Again, the aortic valve should be replaced in the case of QAV with special attention being paid to myocardial protection, to the location of coronary arteries, and of course, to the conduction bundle.

Aneurysmal ascending aortic dilatation is another condition found to be concurrent with QAV and needing to be given special attention [8]. As is well known, aneurysmal ascending aortic dilatation is commonly associated with the bicuspid aortic valve, which is the most frequent abnormality of the aortic valve. However, concomitant aneurysmal ascending aortic dilatation (as in our case, and as also described by other reports) might occasionally be found in future patients with QAV. Aberrant disruption of the medial layer of the aortic wall may cause aneurysmal ascending aortic dilatation [9]. A secondary change of the ascending aorta resulting from a dysfunctional form of QAV is also a possible mechanism for the manifestation of aneurysmal ascending aortic dilatation. Unlike up-to-date surgical guidelines for aortic dilatation in patients with bicuspid aortic valves [10], there is no clinically recommended threshold of aortic dilatation that would justify surgical correction in patients with QAV. We performed supracoronary ascending aorta replacement in accordance with standard recommendation for aortic surgery in cases involving a bicuspid aortic valve. However, a vast data of the clinicopathologic course of aneurysmal ascending aortic dilatation in cases with QAV should be gathered to clarify surgical intervention.

## Conclusions

Current diagnostic technology with its enabling of the performance of high-resolution, noninvasive imaging studies, has given us thorough information about cardiac structural abnormalities, especially in the period of preoperative work-up. However, as reported in the literature, most cases of QAV have still been diagnosed intraoperatively. Therefore, physicians should be fully aware of the anatomical variations of the QAV and the surgical cautions for aortic valve replacement as the role of treatment, just in case of QAV unexpectedly detected during surgery.

## Additional file

Additional file 1: Transthoracic echocardiography showing a quadricuspid aortic valve with an ‘X-shaped’ commissural contour in diastole and central aortic regurgitation resulting from coaptation failure. (MP4 1344 kb)

## Abbreviations

LM: Left main coronary artery
LV: Left ventricle
QAV: Quadricuspid aortic valve
RCA: Right coronary artery
